# Supplementary material for: Antigen glycosylation regulates efficacy of CAR T cells targeting CD19
Source: Nat Commun. 2022 Jun 11;13:3367. doi: 10.1038/s41467-022-31035-7 (PMC9188573; doi:10.1038/s41467-022-31035-7)
Supplement: Supplementary file 1 — Supplementary Information [file 41467_2022_31035_MOESM1_ESM.pdf]

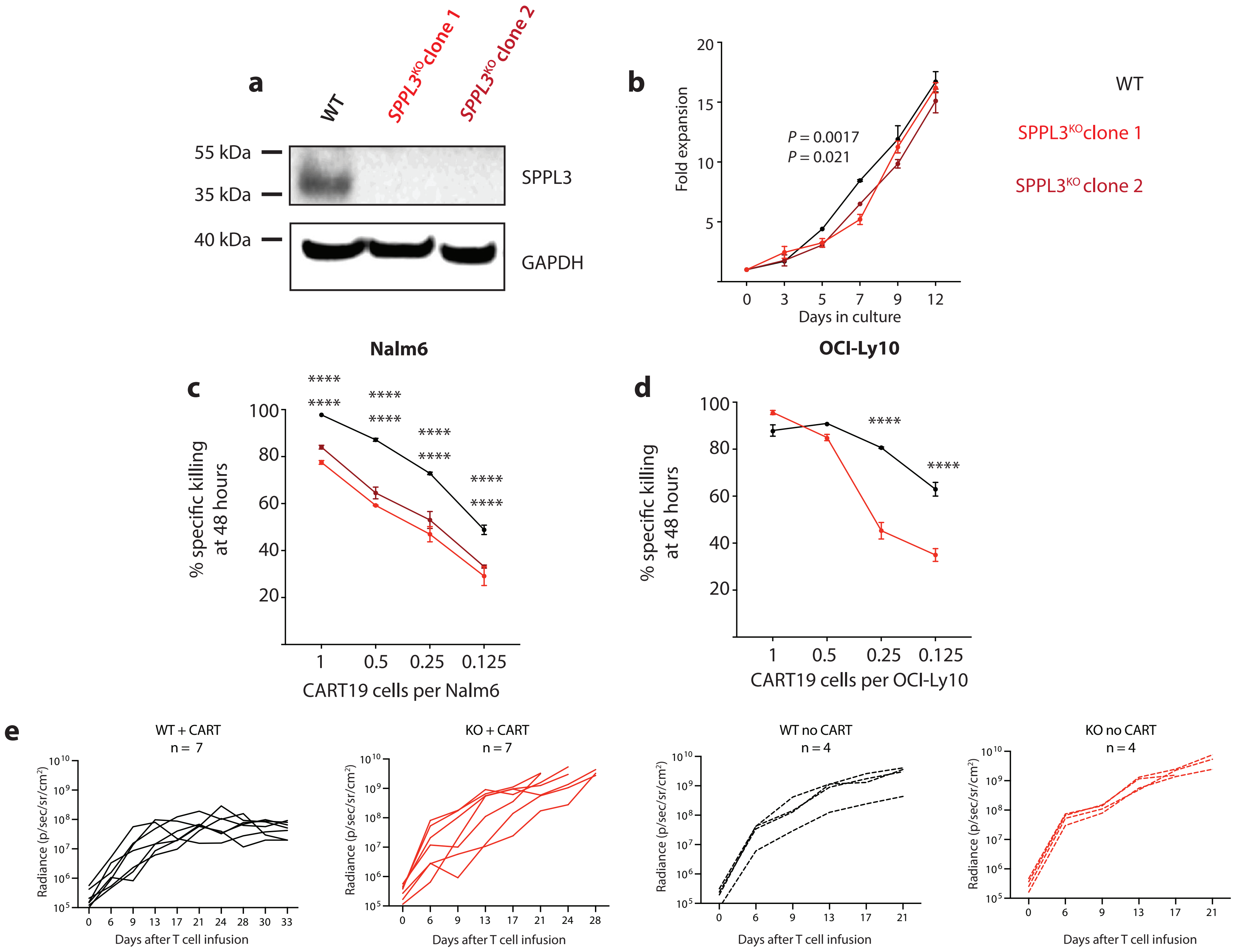

Supplementary Figure 1 | a, Western blot of lysates from WT or SPPL3^{KO} Nalm6 probed for SPPL3. Representative of n=5 individual experiments. b, Fold expansion of WT or SPPL3^{KO} Nalm6 over time. Statistical values indicate significance for clone 1 (above) and clone 2 (below) relative to wild-type. Representative data from n=2 independent experiments. c, Survival of Nalm6 and d, OCI-Ly10 48 hours after combination with CART19 (E:T ratio 0.25:1). Representative data from n = 5 individual experiments. e, Disease burden over time from individual mice. Statistical analysis using two-way ANOVA with Bonferroni correction for multiple comparisons. Error bars reflect mean +/- standard error of the mean (s.e.m.). ****P<0.0001. Source Data are provided in the Supplementary Information File.

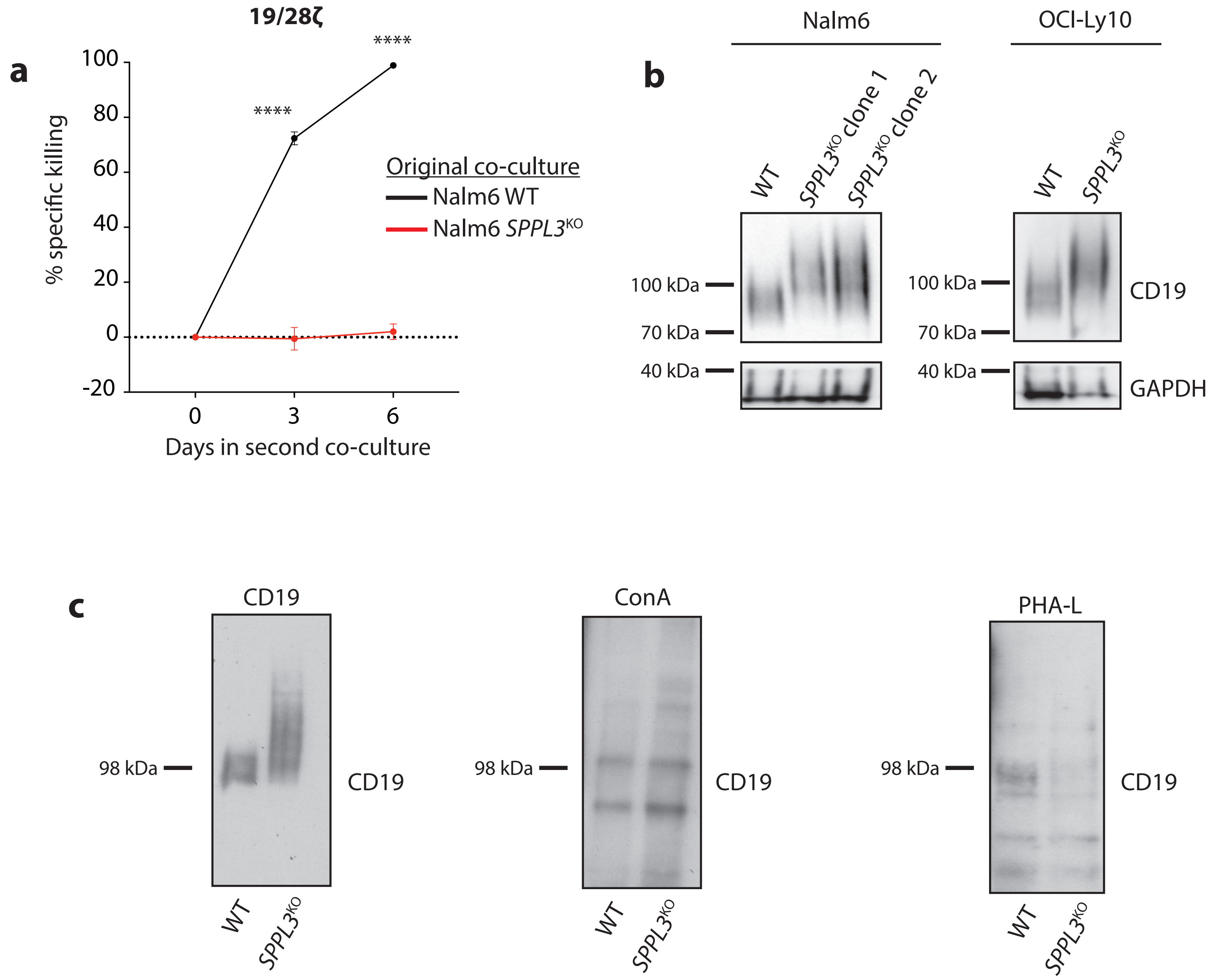

Supplementary Figure 2 | a, Survival of WT Nalm6 over time after combination with T cells bearing a CD19-targeted CAR with a CD28 costimulatory domain. Representative on an individual experiment. b, Western blot of lysates from WT or $SPPL3^{KO}$ Nalm6 and OCI-Ly10 cells probed for CD19. Protein electrophoresis was performed on a 15% polyacrylamide gel. Representative of n=3 individual experiments. c, Lectin blots of immunoprecipitated CD19 from WT or $SPPL3^{KO}$ Nalm6. Lysates were proved with either anti-CD19 antibody (left), concanavalin A (ConA, middle) or phytohaemagglutinin-L (PHA-L, right). Representative data from n=2 experiments. Source Data are provided in the Supplementary Information File.

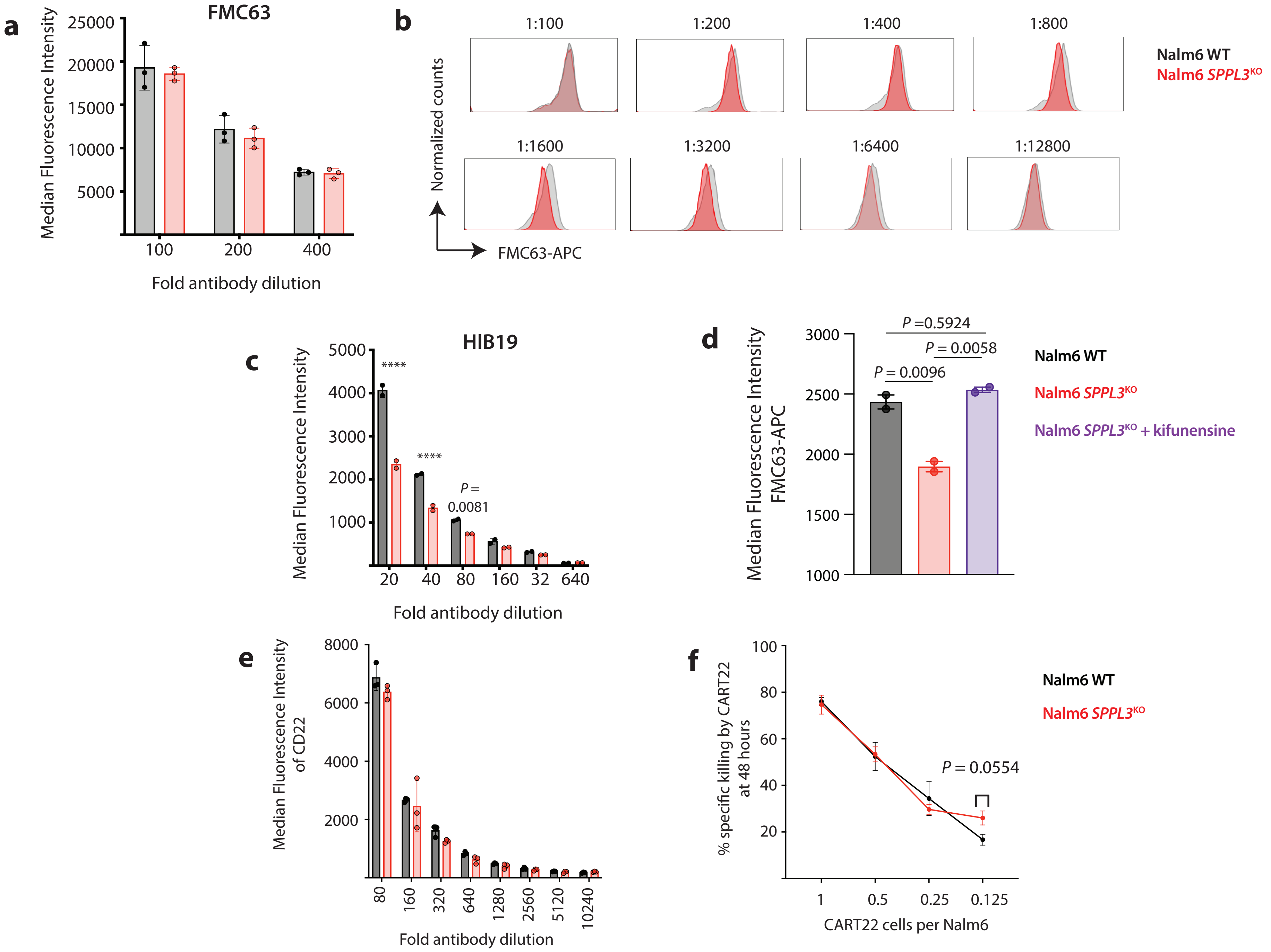

Supplementary Figure 3 | a, Median Fluorescence Intensity of CD19 as detected by FMC63-APC on the surface of WT or SPPL3^{KO} Nalm6 cells. b, Representative histograms of FMC63-APC binding at varying antibody dilutions. c, Median Fluorescence Intensity of CD19 as detected by HIB19-PE on the surface of WT or SPPL3^{KO} Nalm6 cells. d, Median Fluorescence Intensity of CD19 as detected by FMC63-APC on the surface of WT, untreated SPPL3^{KO} Nalm6 or SPPL3^{KO} Nalm6 treated with kifunensine. e, Median Fluorescence Intensity of CD22 on the surface of WT or SPPL3^{KO} Nalm6 cells. a, b, c, e: representative of n=4 individual experiments. d: representative of n=2 individual experiments. f, Survival of WT or SPPL3^{KO} Nalm6 cells 48 hours after combination with CART22 (E:T ratio 0.25:1). Representative data from n=3 individual experiments with distinct donor T cells. Error bars reflect mean +/- standard error of the mean (s.e.m.). ** P <0.01, *** P <0.001, **** P <0.0001 by two-way ANOVA with Bonferroni correction for multiple comparisons. Source Data are provided in the Supplementary Information File.

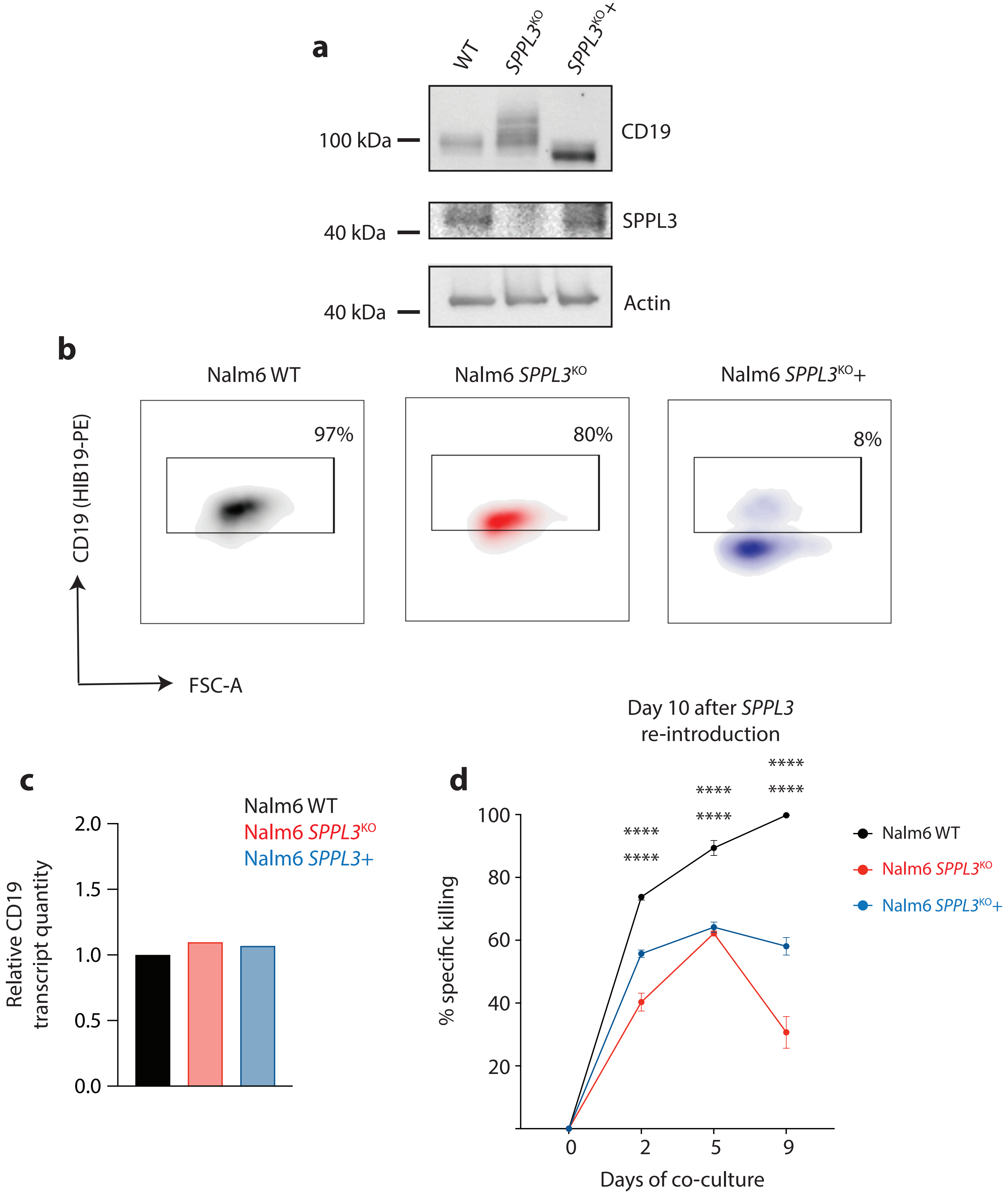

Supplementary Figure 4 | a, Western blot cellular lysates from WT, SPPL3^{KO} or SPPL3^{KO} with over-expression of SPPL3 (SPPL3^{KO}+) Nalm6 cells probed for CD19 performed 5 days after engineering. Representative of an individual experiment. b, Representative flow cytometry plots of CD19 expression on WT, SPPL3^{KO} or SPPL3^{KO}+ Nalm6 as detected by HIB19-PE on day 21 after re-introduction of SPPL3. c, Quantitative RT-PCR for CD19 transcripts from WT, SPPL3^{KO} or SPPL3^{KO} Nalm6 performed several weeks after engineering. d, Survival of WT, SPPL3^{KO} or SPPL3^{KO}+ Nalm6 over time when established on day 10 after SPPL3 re-introduction. Representative data from n=2 experiments with independent T cell donors. Error bars reflect mean +/- standard error of the mean (s.e.m.). * P <0.05, ** P <0.01, *** P <0.001, **** P <0.0001 by two-way ANOVA with Bonferroni correction for multiple comparisons. Statistical annotations reflect differences between WT and SPPL3^{KO} (upper) or WT and SPPL3^{KO}+ Nalm6 (lower). Source Data are provided in the Supplementary Information File.

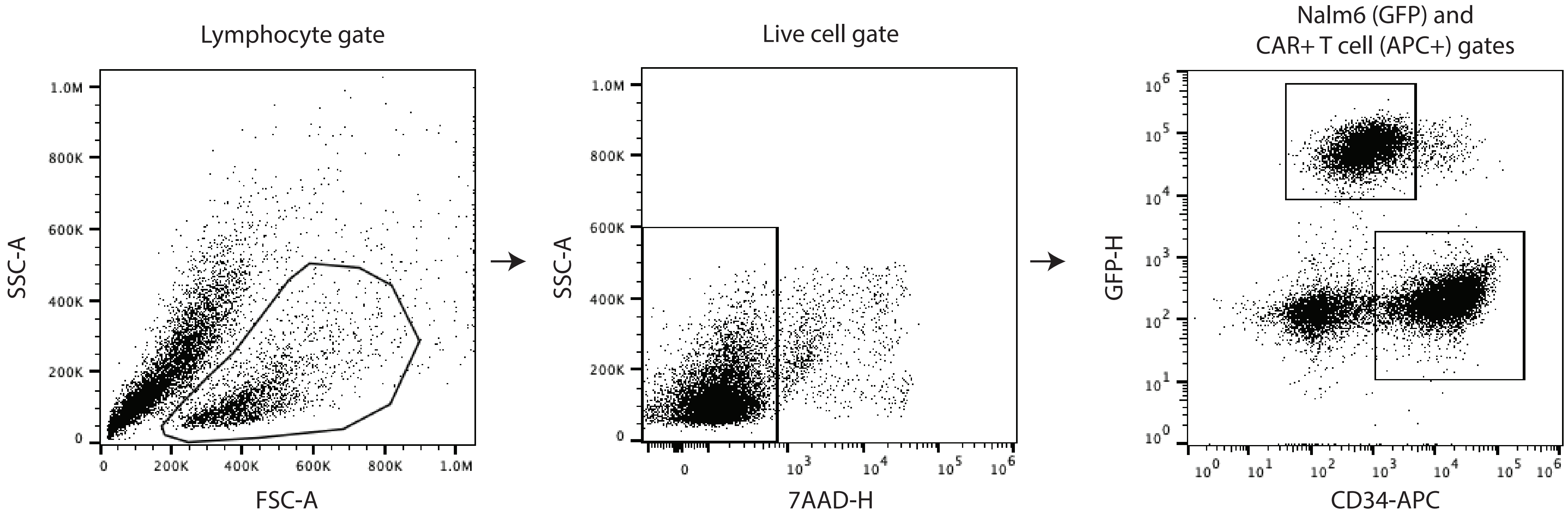

Supplementary Figure 5 | Flow cytometry gating strategy. Applies to data presented in Figures 1c-d, 2b-g and 2i, 3c, 3e and 3g, 4b-c, 5a and 5c, Supplementary Figures 1b-d, 2a, 3a-f, 4b and 4d.
