# Supplementary material for: Antigen glycosylation regulates efficacy of CAR T cells targeting CD19
Source: Nat Commun. 2022 Jun 11;13:3367. doi: 10.1038/s41467-022-31035-7 (PMC9188573; doi:10.1038/s41467-022-31035-7)
Supplement: Supplementary file 2 — Reporting Summary [file 41467_2022_31035_MOESM2_ESM.pdf]

nature portfolio

Corresponding author(s): Nathan Singh, MD

Last updated by author(s): Sep 14, 2021

Reporting Summary

Nature Portfolio wishes to improve the reproducibility of the work that we publish. This form provides structure for consistency and transparency in reporting. For further information on Nature Portfolio policies, see our Editorial Policies and the Editorial Policy Checklist.

Statistics

For all statistical analyses, confirm that the following items are present in the figure legend, table legend, main text, or Methods section.

n/a	Confirmed	
☐	☒	The exact sample size (*n*) for each experimental group/condition, given as a discrete number and unit of measurement
☐	☒	A statement on whether measurements were taken from distinct samples or whether the same sample was measured repeatedly
☐	☒	The statistical test(s) used AND whether they are one- or two-sided<br>*Only common tests should be described solely by name; describe more complex techniques in the Methods section.*
☒	☐	A description of all covariates tested
☐	☒	A description of any assumptions or corrections, such as tests of normality and adjustment for multiple comparisons
☐	☒	A full description of the statistical parameters including central tendency (e.g. means) or other basic estimates (e.g. regression coefficient) AND variation (e.g. standard deviation) or associated estimates of uncertainty (e.g. confidence intervals)
☐	☒	For null hypothesis testing, the test statistic (e.g. *F*, *t*, *r*) with confidence intervals, effect sizes, degrees of freedom and *P* value noted<br>*Give P values as exact values whenever suitable.*
☒	☐	For Bayesian analysis, information on the choice of priors and Markov chain Monte Carlo settings
☒	☐	For hierarchical and complex designs, identification of the appropriate level for tests and full reporting of outcomes
☒	☐	Estimates of effect sizes (e.g. Cohen's *d*, Pearson's *r*), indicating how they were calculated

Our web collection on statistics for biologists contains articles on many of the points above.

Software and code

Policy information about availability of computer code

Data collection	No code used
Data analysis	GraphPad Prism v9, FlowJo v9, TIDE: Tracking of Indels by DEcomposition, Spectral Imaging Instruments AURA, FASTQC v0.11.12, Bowtie v1.2.2, Benchling and UCSF Chimera were used to analyze the data.

For manuscripts utilizing custom algorithms or software that are central to the research but not yet described in published literature, software must be made available to editors and reviewers. We strongly encourage code deposition in a community repository (e.g. GitHub). See the Nature Portfolio guidelines for submitting code & software for further information.

Data

Policy information about availability of data

All manuscripts must include a data availability statement. This statement should provide the following information, where applicable:
- Accession codes, unique identifiers, or web links for publicly available datasets
- A description of any restrictions on data availability
- For clinical datasets or third party data, please ensure that the statement adheres to our policy

Guide library sequencing data are available from Gene Expression Omnibus (GEO) using the accession number GSE130663 (https://www.ncbi.nlm.nih.gov/geo/query/acc.cgi?acc=GSE130663). Sequences used for predictive modeling and analysis were derived from the Protein Database entry 6AL5 (https://www.rcsb.org/structure/6al5). The remaining data are available with the Article, Supplementary Information or Source Data File.

Field-specific reporting

Please select the one below that is the best fit for your research. If you are not sure, read the appropriate sections before making your selection.

[x] Life sciences [ ] Behavioural & social sciences [ ] Ecological, evolutionary & environmental sciences

For a reference copy of the document with all sections, see nature.com/documents/nr-reporting-summary-flat.pdf

Life sciences study design

All studies must disclose on these points even when the disclosure is negative.

Sample size	No sample size calculations were made for the studies described, but sample sizes were chosen based on convention for the field. In accordance with standard practice, biological variability was controlled by repeating studies using multiple independent donor cells and performed in technical replicates.
Data exclusions	No data were excluded.
Replication	Biological replicates were included for all studies (between 2 and 5) using human T cells unless otherwise indicated and experiments were performed in technical triplicate. All attempts at replication were successful for the data presented here.
Randomization	Animals were randomized to treatment groups to ensure consistent initial disease burden. For all in vitro studies, samples were allocated randomly.
Blinding	No blinding was performed for in vitro studies. For animal studies data collection was conducted in a blinded manner.

Reporting for specific materials, systems and methods

We require information from authors about some types of materials, experimental systems and methods used in many studies. Here, indicate whether each material, system or method listed is relevant to your study. If you are not sure if a list item applies to your research, read the appropriate section before selecting a response.

Materials & experimental systems

n/a	Involved in the study	
[ ]	[x]	Antibodies
[ ]	[x]	Eukaryotic cell lines
[x]	[ ]	Palaeontology and archaeology
[ ]	[x]	Animals and other organisms
[x]	[ ]	Human research participants
[x]	[ ]	Clinical data
[x]	[ ]	Dual use research of concern

Methods

n/a	Involved in the study	
[x]	[ ]	ChIP-seq
[ ]	[x]	Flow cytometry
[x]	[ ]	MRI-based neuroimaging

Antibodies

Antibodies used	Flow cytometry: CD3 (clone OKT3, BD Biosciences 555342, diluted 1:50), PD-1 (clone EH12.2H7, BioLegend, 329928, diluted 1:80), Tim3 (clone 7D3, BD Biosciences 565566, diluted 1:100), CD22 (clone HIB22, BD Biosciences 562860, diluted 1:100), CD19 (clone FMC63, Novus Biologicals 52716, diluted 1:80-1:32000; clone HIB19, BD Biosciences 555413, diluted 1:50), CD107a-PECy7 (clone H4A3, Biolegend 328607, diluted 1:100), CD34 APC (BD 555824, diluted 1:50).<br><br>Western Blot: SPPL3 (EMD Millipore #MABS1910), CD19 (#3574), CD22 (#67434), Actin (#4970) or GAPDH (#2118, all from CellSignaling, all diluted 1:1000); secondary anti-rabbit (CellSignaling #7074, diluted 1:5000) and anti-mouse (CellSignaling #7076, diluted 1:5000) HRP-linked antibodies.
Validation	All antibodies were validated for use in vitro and on animal tissue by the commercial provider.

Eukaryotic cell lines

Policy information about cell lines

Cell line source(s)	All cell lines were originally obtained from ATCC. Lines used were Nalm6, OCI-Ly10 and HEK293T.
Authentication	Parent cell lines were genotyped by short tandem repeat (STR) analysis every six months, or after any genetic modification to ensure identity.

Mycoplasma contamination	All cell lines were tested for mycoplasma contamination every six months and confirmed negative.
Commonly misidentified lines (See ICLAC register)	None used.

Animals and other organisms

Policy information about studies involving animals; ARRIVE guidelines recommended for reporting animal research

Laboratory animals	NSG mice were obtained from Jackson Laboratories. Mice were mixed male and female of 6-12 weeks age.
Wild animals	No wild animals were used.
Field-collected samples	No field animals were collected for this study.
Ethics oversight	Animal studies were approved by the Washington University Institutional Animal Care and Use Committee, Assurance #A-3381-01.

Note that full information on the approval of the study protocol must also be provided in the manuscript.

Flow Cytometry

Plots

Confirm that:

- [x] The axis labels state the marker and fluorochrome used (e.g. CD4-FITC).
- [x] The axis scales are clearly visible. Include numbers along axes only for bottom left plot of group (a 'group' is an analysis of identical markers).
- [x] All plots are contour plots with outliers or pseudocolor plots.
- [x] A numerical value for number of cells or percentage (with statistics) is provided.

Methodology

Sample preparation	Cells, either lines or primary human T cells, were stained in antibodies diluted in PBS + 3% FBS for 20 minutes, followed by two washes with PBS+3% FBS.
Instrument	Acquisition was performed on a ThermoFisher Attune NxT
Software	FlowJo v9 was used to analyze all data.
Cell population abundance	Cells were not sorted for these studies.
Gating strategy	Cells were first gated morphologically on FSC-A/SSC-A to identify live cells, then on 7AAD to exclude dead cells. Cells were then evaluated for expression of each given protein.

- [x] Tick this box to confirm that a figure exemplifying the gating strategy is provided in the Supplementary Information.
